# Silencing of Long Noncoding RNA LINC00324 Interacts with MicroRNA-3200-5p to Attenuate the Tumorigenesis of Gastric Cancer via Regulating BCAT1

**DOI:** 10.1155/2020/4159298

**Published:** 2020-08-13

**Authors:** Shuang Wang, Yuanyuan Cheng, Pingping Yang, Guang Qin

**Affiliations:** Department of Medical Oncology I, Taian City Central Hospital, No. 29, Longtan Road, Taian City, Shandong Province 271000, China

## Abstract

**Purpose:**

This study was aimed at exploring the effect of long noncoding RNA LINC00324 (LINC00324) on gastric cancer (GC) and the potential molecular mechanisms.

**Methods:**

The expression of LINC00324 and miR-3200-5p in GC tissues and cells was detected by qRT-PCR. LINC00324 was silenced in GC cells by transfection of si-LINC00324. Then, the proliferation, migration, and invasion of GC cells were analyzed by MTT, wound healing, and transwell assays, respectively. The interactions between LINC00324 and miR-3200-5p and between miR-3200-5p and BCAT1 were determined by a dual-luciferase reporter and/or RNA pull-down assay.

**Results:**

The expression of LINC00324 was upregulated in GC cells and tissues, but miR-3200-5p was downregulated. Silencing of LINC00324 inhibited the proliferation, migration, and invasion of GC cells. LINC00324 directly targeted miR-3200-5p, and miR-3200-5p directly targeted BCAT1. si-LINC00324 negatively regulated BCAT1 expression via binding to miR-3200-5p. Furthermore, silencing of LINC00324 reversed the promoting effects of BCAT1 on the proliferation, migration, and invasion of GC cells.

**Conclusion:**

Silencing of LINC00324 inhibited the proliferation, migration, and invasion of GC cells through regulating the miR-3200-5p/BCAT1 axis.

## 1. Introduction

Gastric cancer (GC), also known as stomach cancer, is the third most frequent malignancy worldwide [1]. Less than 30% of GC patients survive for more than five years after making a definite diagnosis [2]. It is difficult to distinguish benign peptic ulcer in most patients with early GC [3]. The aggressive proliferation and migration of GC cells are critical factors responsible for the poor survival of patients with GC [4]. Therefore, it is essential to explore the potential targets for the treatment of GC.

Long noncoding RNAs (lncRNAs) are noncoding RNA transcripts, taking part in the occurrence and progression of diverse digestive organ cancers including esophageal cancer [5], pancreatic cancer [6], and colorectal cancer [7]. lncRNA LINC00324 (LINC00324) belongs to the intragenic lncRNAs, which is located in 17p13.1 [8]. Recently, some studies have indicated that LINC00324 is involved in cancer progression. For example, LINC00324 increases the proliferation and migration of osteosarcoma cells [9]. LINC00324 promotes the tumorigenesis of lung adenocarcinoma [8]. However, the role of LINC00324 in the occurrence and progression of GC is unclear.

MicroRNAs (miRNAs) are small noncoding RNAs which mediate target genes by interacting with the 3′ untranslated regions (3′ UTRs) [10]. Some miRNAs exhibit an obvious anticancer effect on GC. For instance, miR-218 acts as a tumor-suppressive miRNA in GC via targeting WASF3 expression [11]. Overexpression of miR-338 attenuates the tumorigenesis of GC through regulating NRP1 expression [12]. MiR-1254 overexpression increases cell proliferation, migration, and invasion in GC via inhibiting Smurf1[13]. Zhang et al. have displayed that silencing of lncRNA PEG10 inhibits the occurrence and progression of GC by regulating miR-3200 expression [14]. However, the specific regulatory relationship between LINC00324 and miR-3200-5p remains undefined in GC.

Branched-chain amino acid transaminase 1 (BCAT1) is a cytosolic BCAT isozyme expressed in specific tissues [15]. BCAT1 takes part in the progression of several malignancies, including glioblastoma [16], hepatocellular carcinoma [17], and nasopharyngeal carcinoma [18]. In addition, Xu et al. have reported that BCAT1 acts as a valuable prognostic marker in GC [19]. Nevertheless, the potential regulatory mechanism of LINC00324 involving BCAT1 is still unknown in GC.

Here, the effects of LINC00324 on the proliferation, migration, and invasion of GC cells were evaluated. Then, we investigated whether miR-3200-5p is a downstream target of LINC00324 and the association between miR-3200-5p and BCAT1. This study may reveal a hopeful therapeutic target for GC and the underlying mechanisms for the treatment of GC.

## 2. Methods

### 2.1. Tissue Samples

In total, 60 GC patients (33 males and 27 females, 52.66 ± 9.70 years old) were collected from our hospital between April 2016 and May 2018. Fresh GC tissues and adjacent normal tissues were obtained from GC patients who underwent surgery at our hospital. The tissues were frozen in liquid nitrogen immediately after resection and stored at -80°C for future use. This study was permitted by the ethics committee of Taian City Central Hospital, and written informed consent was obtained from all the patients.

### 2.2. Cell Culture

Three human GC cell lines, AGS, MGC803, and MKN-45, and the human normal gastric epithelial cell line GES-1 were obtained from the American Type Culture Collection. Cells were cultured in Dulbecco's Modified Eagle's Medium (DMEM, catalog # 30030, Invitrogen, Carlsbad, CA, USA) containing 10% fetal bovine serum (FBS, catalog # 16000-044, Invitrogen) at 37°C with 5% CO_2_.

### 2.3. Cell Transfection

The siRNA-negative control (si-NC), si-LINC00324-1, si-LINC00324-2, mimics NC, miR-3200-5p mimics, miR-3200-5p inhibitor, inhibitor NC, pcDNA3.1-NC (pcDNA-NC), and pcDNA3.1 BCAT1 (pcDNA-BCAT1) were purchased from Shanghai GenePharma (Shanghai, China). MGC803 and MKN-45 cells grown to 80% confluence were transfected with these above agents using the Lipofectamine 3000 reagent (catalog # L3000075, Invitrogen). MGC803 and MKN-45 cells were divided into si-NC, si-LINC00324-1, si-LINC00324-2, mimics NC, miR-3200-5p mimics, inhibitor NC, miR-3200-5p inhibitor, si-NC+pcDNA-NC, si-NC+pcDNA-BCAT1, and si-LINC00324+pcDNA-BCAT1 groups. Cells without transfection were considered the control or blank groups.

### 2.4. Quantitative Real-Time Polymerase Chain Reaction (qRT-PCR)

Total RNA was extracted from tissues and cells using the TRIzol reagent (catalog # 10296028, Invitrogen). RNA was quantified with a NanoDrop ND-1000 spectrophotometer (Wilmington, Delaware, USA). A total of 300 ng RNA was reverse-transcribed into cDNA using a PrimeScript RT reagent kit (catalog # RR037A v.0610, Takara, Otsu, Japan). PCR reaction was performed on the ABI 7500HT Fast Real-Time PCR System (Applied Biosystems, Waltham, MA, USA) with the following conditions: 95°C for 3 min and 40 cycles of 95°C for 15 s and 60°C for 30 s. GAPDH, U6, and *β*-actin were used for the normalization of LINC00324, miR-3200-5p, and BCAT1, respectively. The primer sequences are shown in Table 1. The relative expression level was calculated by the 2^-*ΔΔ*Ct^ method.

### 2.5. MTT Assay

MGC803 and MKN-45 cells were seeded into 96-well plates (2 × 10^3^ cells/well). After 24, 48, 72, and 96 h of culturing, 20 *μ*L MTT (catalog # M5655, Sigma, St. Louis, MO, USA) was added into each well, and the cells were incubated for 4 h at 37°C with 5% CO_2_. After the supernatant was discarded, 150 *μ*L dimethyl sulfoxide (DMSO, catalog # D2650, Sigma) was added to dissolve the formazan. The absorbance at 450 nm was measured using a microplate reader (Thermo Fisher Scientific, Waltham, MA, USA).

### 2.6. Wound Healing Assay

When MGC803 and MKN-45 cells were cultured at 80% confluence, an artificial scratch was created using a 10 *μ*L pipette tip. Cells were then incubated for 48 h and observed under an inverted microscope (Olympus, Tokyo, Japan). The wound healing rate was calculated by the fraction of cell coverage across the line.

### 2.7. Transwell Assay

The transwell assay was used to determine the cell invasion using a transwell chamber (8 *μ*m pore size) (catalog # 354480, Corning Inc., Corning, NY, USA). MGC803 and MKN-45 cells (2 × 10^5^) in serum-free medium were added to the upper chamber precoated with Matrigel (catalog # 354234, BD Biosciences, San Jose, CA, USA). Roswell Park Memorial Institute- (RPMI-) 1640 (catalog # 31800022, GIBCO, Erie, NY, USA) medium with 10% FBS (Invitrogen) was added to the lower chamber. After 48 h of incubation at 37°C, cells were removed from the upper chamber with a cotton swab, and cells in the lower chamber were fixed in methanol and stained with 0.5% crystal violet for 2 min. Five random fields were photographed.

### 2.8. Dual-Luciferase Reporter Assay

The potential binding sites of miR-3200-5p and LINC00324 or miR-3200-5p and BCAT1 were predicted by Starbase or TargetScan, respectively. LINC00324 and BCAT1 with WT or MUT miR-3200-5p-binding sites were generated and subcloned into the psiCHECK-2 vector (catalog # C8021, Promega, Madison, WI, USA). The vectors were then cotransfected with mimics NC, miR-3200-5p mimics, and miR-3200-5p mimics+LINC00324 into MGC803 and MKN-45 cells using Lipofectamine 3000 (Invitrogen) for 48 h. The luciferase activity was detected by a dual-luciferase reporter gene assay system (Promega).

### 2.9. RNA Pull Down

Bio-LINC00324-Wt, Bio-LINC00324-Mut, and Bio-NC (GenePharma) were transfected into GC cells (MGC803 and MKN-45) using Lipofectamine 3000 (Invitrogen). After culturing for 48 h, cells were incubated with Dynabeads M-280 streptavidin beads (catalog # 60210, Invitrogen) for 1 h. The qRT-PCR assay was employed to assess the enrichment of BCAT1.

### 2.10. Statistical Analysis

Statistical analysis was performed using SPSS 23.0 (SPSS Inc., Chicago, IL, USA). Data were presented as mean ± standard deviation (SD). The differences among multiple groups were analyzed by one-way ANOVA followed by Tukey's multiple comparison test. The differences between two groups were assessed using Student's *t*-test. The correlation was determined by the Pearson correlation analysis. A *P* value < 0.05 was considered statistically significant.

## 3. Results

### 3.1. The Expression of LINC00324 Was Increased in GC Tissues and Cells

**Figure 1:**
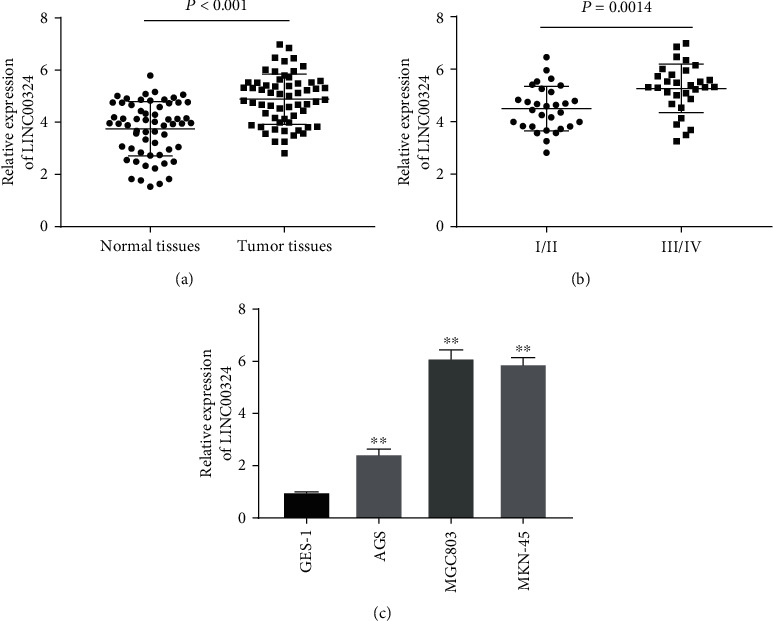
The expression of LINC00324 was increased in gastric cancer (GC) tissues and cells. (a) The expression of LINC00324 in tumor tissues and adjacent normal tissues of patients with GC was detected by qRT-PCR. (b) The expression of LINC00324 in patients with GC at TNM I/II and TNM III/IV was detected by qRT-PCR. (c) The expression of LINC00324 in the human normal gastric epithelial cell line GES-1 and GC cell lines (AGS, MGC803, and MKN-45) was detected by qRT-PCR. ^∗∗^*P* < 0.01 vs. GES-1.

qRT-PCR was performed to confirm whether LINC00324 is differently expressed in GC tissues. The results showed that the expression of LINC00324 in tumor tissues was markedly increased compared with that in adjacent normal tissues in patients with GC (*P* < 0.001) (Figure 1(a)). Additionally, the expression of LINC00324 in tumors at TNM III/IV was markedly increased compared with that in tumors at TNM I/II (*P* < 0.01) (Figure 1(b)). The correlation between LINC00324 expression and clinical characteristics of patients with GC was then analyzed. The results showed that LINC00324 expression was positively correlated with tumor size (*P* < 0.05), lymphoma metastasis (*P* < 0.01), and TNM stage in GC patients (*P* < 0.01). However, LINC00324 expression had no connection with age, gender, and histological grade in GC patients (*P* > 0.05) (Table 2). qRT-PCR was further performed to detect the expression of LINC00324 in GES-1, AGS, MGC803, and MKN-45 cells. The results showed that the expression of LINC00324 in AGS, MGC803, and MKN-45 cells was significantly increased compared with that in GES-1 cells (*P* < 0.01) (Figure 1(c)). MGC803 and MKN-45 cells were used for subsequent assays due to relatively high expression of LINC00324.

### 3.2. Silencing of LINC00324 Inhibited the Proliferation, Migration, and Invasion of GC Cells

**Figure 2:**
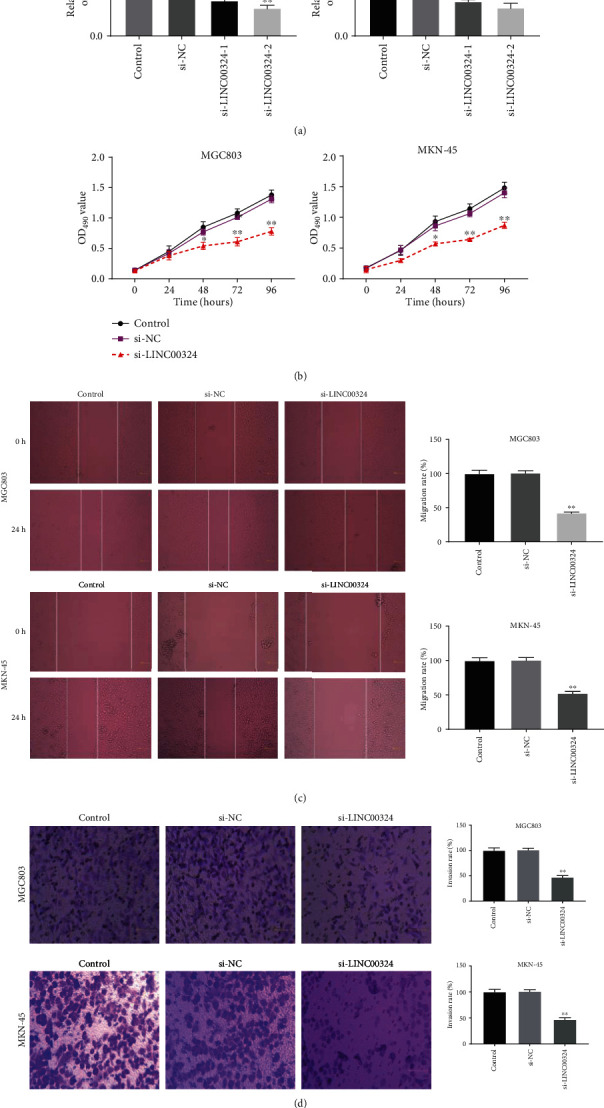
Silencing of LINC00324 inhibited the proliferation, migration, and invasion of gastric cancer (GC) cells. (a) The expression of LINC00324 in MGC803 and MKN-45 cells was detected by qRT-PCR. (b) The OD_450_ value of MGC803 and MKN-45 cells was detected by the MTT assay. (c) The migration rate of MGC803 and MKN-45 cells was detected by the wound healing assay. (d) The invasion rate of MGC803 and MKN-45 cells was detected by the transwell assay. ^∗^*P* < 0.05, ^∗∗^*P* < 0.01 vs. control.

To evaluate the effects of LINC00324 on the proliferation, migration, and invasion of GC cells, LINC00324 was silenced by the transfection of si-LINC00324-1 and si-LINC00324-2 in MGC803 and MKN-45 cells. The expression of LINC00324 in MGC803 and MKN-45 cells in the si-LINC00324-1 and si-LINC00324-2 groups was significantly downregulated compared with that in the control group (*P* < 0.01) (Figure 2(a)). si-LINC00324-2 was used for subsequent functional assays due to its relatively high silencing efficiency. The MTT assay showed that si-LINC00324 significantly decreased the OD_450_ value of MGC803 and MKN-45 cells at 48, 72, and 96 h postculturing (*P* < 0.05) (Figure 2(b)). The wound healing assay showed that the migration rate of MGC803 and MKN-45 cells in the si-LINC00324 group was markedly decreased compared with that in the control group (*P* < 0.01) (Figure 2(c)). The transwell assay showed that the invasion rate of MGC803 and MKN-45 cells in the si-LINC00324 group was significantly decreased compared with that in the control group (*P* < 0.01) (Figure 2(d)).

### 3.3. LINC00324 Was Negatively Correlated with miR-3200-5p

**Figure 3:**
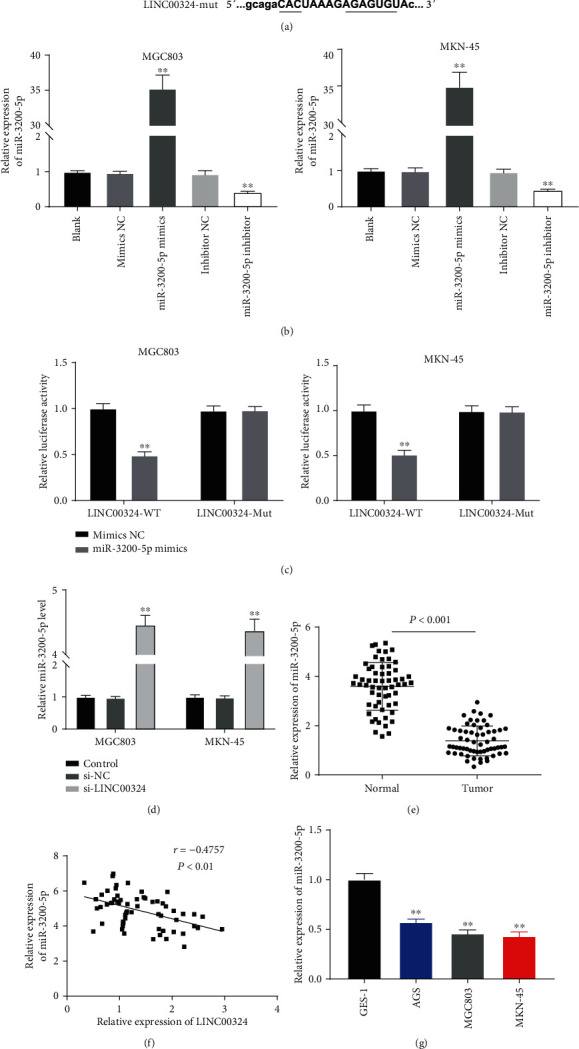
miR-3200-5p was a target of LINC00324. (a) Starbase predicted a binding site between LINC00324 and miR-3200-5p. (b) MGC803 and MKN-45 cells were transfected with mimics NC, miR-3200-5p mimics, inhibitor NC, or miR-3200-5p inhibitor. ^∗∗^*P* < 0.01 vs. blank. (c) Relative luciferase activity in MGC803 and MKN-45 cells was measured by the dual-luciferase reporter assay. ^∗∗^*P* < 0.01 vs. mimics NC. (d) MGC803 and MKN-45 cells were transfected with si-NC or si-LINC00324. ^∗∗^*P* < 0.01 vs. control. (e) The expression of LINC00324 in tumor tissues and adjacent normal tissues of patients with GC was detected by qRT-PCR. ^∗∗∗^*P* < 0.001 vs. normal tissue. (f) The expression of LINC00324 was negatively correlated with miR-3200-5p. (g) The expression of miR-3200-5p in the human normal gastric epithelial cell line GES-1 and GC cell lines (AGS, MGC803, and MKN-45) was detected by qRT-PCR. ^∗∗^*P* < 0.01 vs. GES-1.

To confirm the downstream mechanism of LINC00324 in GC, we predicted the targets of LINC00324. A binding site of miR-3200-5p on the 3′ UTR of LINC00324 was predicted by Starbase (Figure 3(a)). Subsequently, the interaction between LINC00324 and miR-3200-5p was further determined in GC cells. miR-3200-5p was overexpressed by the transfection of miR-3200-5p mimics and blocked by the transfection of the miR-3200-5p inhibitor in GC cells (*P* < 0.01) (Figure 3(b)). The dual-luciferase reporter assay showed that the relative luciferase activity in MGC803 and MKN-45 cells cotransfected with miR-3200-5p mimics and LINC00324-Wt was markedly decreased compared with that in cells cotransfected with mimics NC and LINC00324-Wt (*P* < 0.01) (Figure 3(c)). In addition, qRT-PCR showed that the expression of miR-3200-5p in MGC803 and MKN-45 cells was significantly increased by LINC00324 silencing (*P* < 0.01) (Figure 3(d)). The expression of miR-3200-5p was then detected in GC tissues. qRT-PCR showed that the expression of miR-3200-5p in tumor tissues was significantly decreased compared with that in adjacent normal tissues in patients with GC (*P* < 0.001) (Figure 3(e)). There was a negative correlation between LINC00324 and miR-3200-5p expression in GC tissues (*N* = 60, *r* = −0.4757, *P* < 0.01) (Figure 3(f)). qRT-PCR was further performed to detect the expression of LINC00324 in GC cells. The results revealed that the expression of miR-3200-5p in AGS, MGC803, and MKN-45 cells was markedly decreased compared with that in GES-1 cells (*P* < 0.01) (Figure 3(g)).

### 3.4. LINC00324 Positively Regulated BCAT1 Expression via Binding to miR-3200-5p

**Figure 4:**
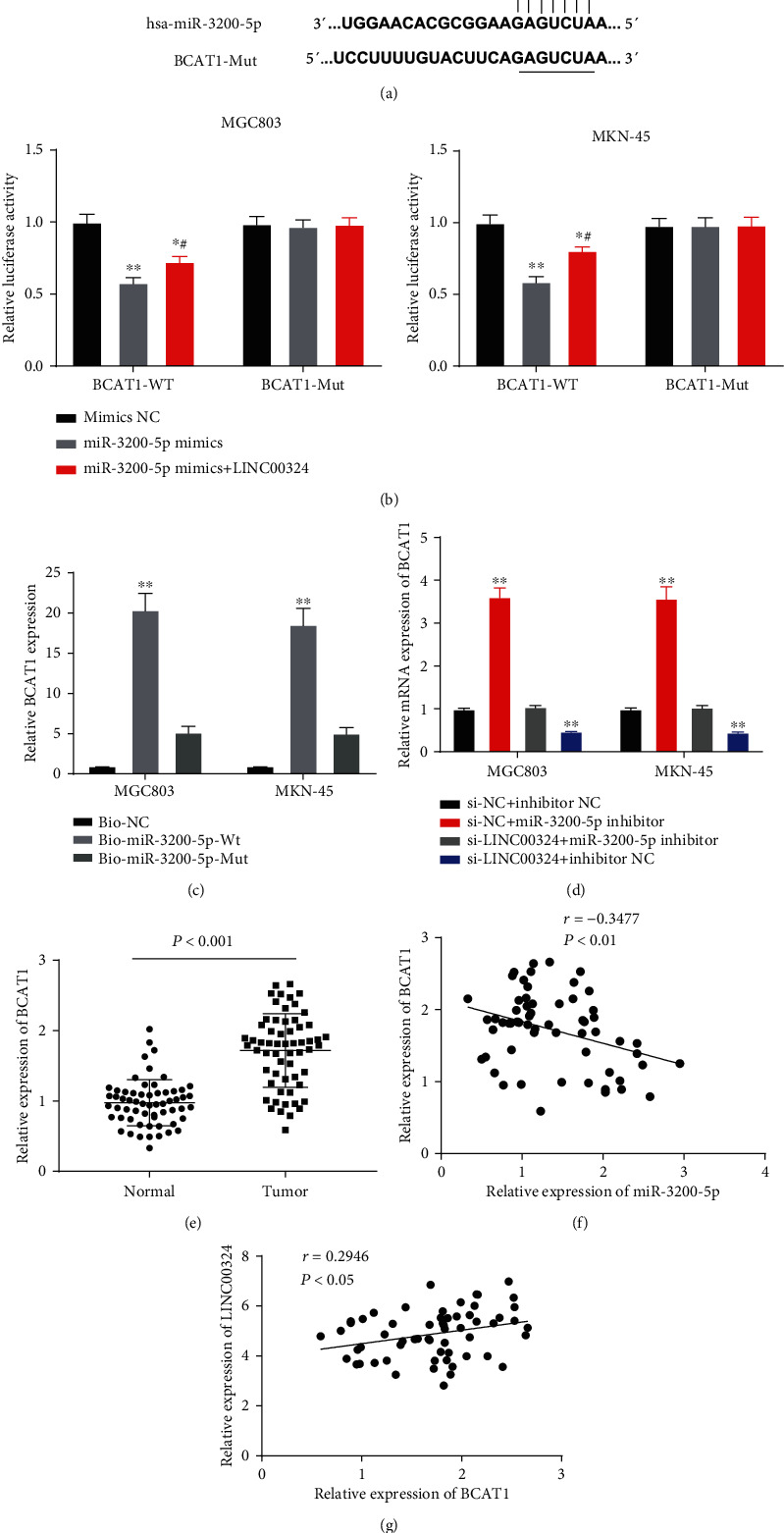
LINC00324 positively regulated BCAT1 expression via binding to miR-3200-5p. (a) TargetScan predicted a binding site between BCAT1 and miR-3200-5p. (b) Relative luciferase activity in MGC803 and MKN-45 cells was measured by the dual-luciferase reporter assay. ^∗^*P* < 0.05, ^∗∗^*P* < 0.01 vs. mimics NC; ^#^*P* < 0.05 vs. miR-3200-5p mimics. (c) The interaction between BCAT1 and miR-3200-5p in MGC803 and MKN-45 cells was detected by the RNA pull-down assay. ^∗∗^*P* < 0.01 vs. Bio-NC. (d) The expression of BCAT1 in MGC803 and MKN-45 cells was detected by qRT-PCR. ^∗∗^*P* < 0.01 vs. si-NC+inhibitor NC and si-LINC00324+miR-3200-5p inhibitor. (e) The expression of BCAT1 in tumor tissues and adjacent normal tissues of patients with GC was detected by qRT-PCR. ^∗∗∗^*P* < 0.001 vs. normal tissue. (f) The expression of BCAT1 was negatively correlated with miR-3200-5p. (g) The expression of BCAT1 was positively correlated with LINC00324.

The downstream target of miR-3200-5p was predicted by TargetScan. A binding site of miR-3200-5p on the 3′ UTR of BCAT1 was predicted (Figure 4(a)). Subsequently, the interactions among LINC00324, miR-3200-5p, and BCAT1 were determined in GC cells. The dual-luciferase reporter assay showed that the relative luciferase activity in MGC803 and MKN-45 cells cotransfected with miR-3200-5p mimics and BCAT1-Wt was significantly decreased compared with that in cells cotransfected with mimics NC and BCAT1-Wt (*P* < 0.01), and overexpression of LINC00324 reversed the reducing effect of miR-3200-5p mimics on the relative luciferase activity (*P* < 0.05) (Figure 4(b)). The RNA pull-down assay showed that BCAT1 was pulled down by Bio-miR-3200-5p-Wt, while Bio-miR-3200-5p-MUT with a mutated binding site of BCAT1 failed to pull down BCAT1 (*P* < 0.01) (Figure 4(c)). In addition, qRT-PCR showed that the expression of BCAT1 was markedly increased by the transfection of the miR-3200-5p inhibitor and decreased by the transfection of si-LINC00324 in MGC803 and MKN-45 cells (*P* < 0.01) (Figure 4(d)). These results indicated that LINC00324 might upregulate BCAT1 expression by binding to miR-3200-5p. Furthermore, the expression of BCAT1 was detected in GC tissues. qRT-PCR showed that the expression of BCAT1 in tumor tissues was markedly increased compared with that in adjacent normal tissues in patients with GC (*P* < 0.001) (Figure 4(e)). There were a negative correlation between BCAT1 and miR-3200-5p expression (*N* = 60, *r* = −0.3477, *P* < 0.01) (Figure 4(f)) and a positive correlation between LINC00324 and BCAT1 expression in GC tissues (*N* = 60, *r* = 0.2946, *P* < 0.05) (Figure 4(g)).

### 3.5. Silencing of LINC00324 Attenuated the Proliferation, Migration, and Invasion of GC Cells via Targeting BCAT1

**Figure 5:**
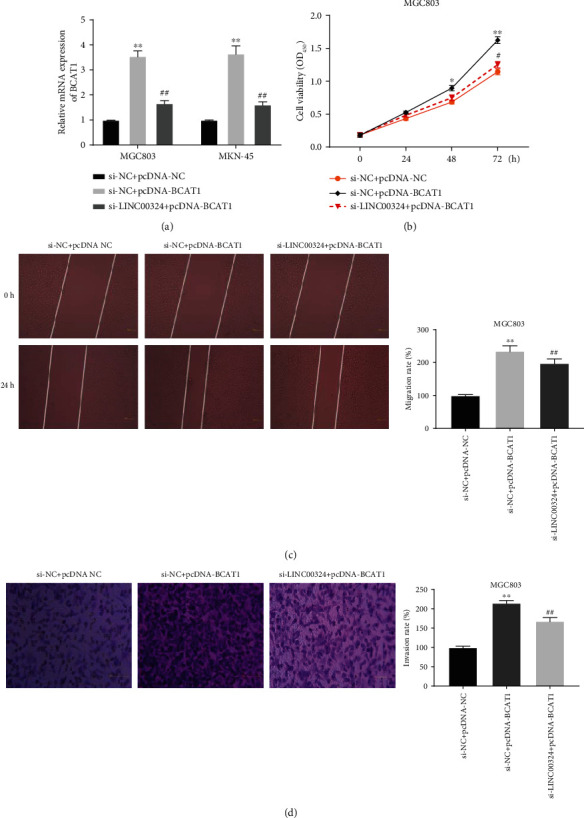
Silencing of LINC00324 attenuated the proliferation, migration, and invasion of gastric cancer (GC) cells via regulating BCAT1. (a) The expression of BCAT1 in MGC803 cells was detected by qRT-PCR. (b) The OD_450_ value of MGC803 cells was detected by the MTT assay. (c) The migration rate of MGC803 cells was detected by the wound healing assay. (d) The invasion rate of MGC803 cells was detected by the transwell assay. ^∗^*P* < 0.05, ^∗∗^*P* < 0.01 vs. si-NC+pcDNA-NC; ^#^*P* < 0.05, ^##^*P* < 0.01 vs. si-LINC00324+pcDNA-BCAT1.

To further investigate the regulatory relationship between LINC00324 and BCAT1, BCAT1 was overexpressed by the transfection of pcDNA-BCAT1 in GC cells (*P* < 0.01). The expression of BCAT1 in the si-LINC00324+pcDNA-BCAT1 group was markedly decreased compared with that in the si-NC+pcDNA-BCAT1 group (*P* < 0.01) (Figure 5(a)). Rescue experiments were then performed to determine whether LINC00324 regulated the proliferation, migration, and invasion of GC cells via targeting BCAT1. The MTT assay showed that the OD_450_ value of MGC803 cells in the si-NC+pcDNA-BCAT1 group at 48 (*P* < 0.05) and 72 h (*P* < 0.01) postculturing was markedly increased compared with that in the si-NC+pcDNA-NC group. Silencing of LINC00324 markedly reversed the promoting effect of BCAT1 on the proliferation of MGC803 cells at 72 h postculturing (*P* < 0.01) (Figure 5(b)). Wound healing and transwell assays showed that the migration and invasion rates of MGC803 cells in the si-NC+pcDNA-BCAT1 group were markedly increased compared with those in the si-NC+pcDNA-NC group (*P* < 0.01). Silencing of LINC00324 markedly reversed the promoting effects of BCAT1 on the migration and invasion of MGC803 cells (*P* < 0.01) (Figures 5(c) and 5(d)).

## 4. Discussion

Abnormal expression of lncRNAs is related to the tumorigenesis of GC [20, 21]. Some lncRNAs, such as Sox2ot [22], ANRIL [23], and H19 [24], are upregulated in patients with GC. In this study, the expression of LINC00324 in tumor tissues was higher than that in adjacent normal tissues in GC patients. This result indicated that LINC00324 plays a promoting role in the tumorigenesis of GC. In addition, the analysis of clinical characteristics displayed that the expression of LINC00324 was correlated with tumor size, lymphoma metastasis, and TNM stage in GC patients. This correlation of LINC00324 with GC was similar to some previously reported lncRNAs. Xu et al. have proven that the expression of lncRNA AC130710 is related to TNM stages and distal metastasis of GC [25]. Liu et al. have suggested that the expression of lncRNA VPS9D1-AS1 is correlated with the tumor size of GC [26]. Our results suggest that LINC00324 may be a valuable biomarker for GC.

Previous researches have confirmed that LINC00324 takes part in cell proliferation, invasion, and migration in cancer. Wu et al. have proven that LINC00324 increases the proliferation and migration of osteosarcoma cells via targeting WDR66 [9]. Pan et al. have shown that LINC00324 has the ability to promote the growth of lung adenocarcinoma through regulating the miR-615-5p/AKT1 axis [8]. Notably, Zou et al. have reported that LINC00324 accelerates the proliferation of GC cells through stabilizing FAM83B expression and binding to HuR [27]. In the present study, si-LINC00324 decreased the proliferation, migration, and invasion of GC cells. The function of LINC00324 in GC was similar to that in the above researches. Our results indicate that silencing of LINC00324 may attenuate the tumorigenesis of GC.

lncRNAs can serve as molecular sponges to competitively regulate miRNAs in GC. For instance, lncRNA-RMRP promotes cell proliferation by acting as a sponge of miR-206 in GC [28]. lncRNA MT1JP accelerates cell proliferation, migration, and invasion through competitively binding to miR-92a-3p in GC [29]. In this study, miR-3200-5p was determined to be a target of LINC00324. The expression of miR-3200-5p was downregulated in GC tissues, which was negatively related to LINC00324. miR-3200 plays a key regulatory role in tumors. Al-Khanbashi et al. have shown that miR-3200 serves as a tumor suppressor in locally advanced breast cancer [30]. Wang et al. have found that miR-3200 attenuates the proliferation of GC cells [31]. Based on the above previous findings, we speculate that silencing of LINC00324 may inhibit the progression of GC by regulating miR-3200-5p.

The expression of BCAT1 is usually upregulated in diverse cancers, such as ovarian cancer [32], breast cancer [33], and GC [19]. Similar to previous studies, we found that the expression of BCAT1 was markedly upregulated in GC tissues. BCAT1 has been proven to be involved in the progression of cancer. For instance, knockdown of BCAT1 has an anticancer effect on nasopharyngeal carcinoma [18]. BCAT1 accelerates the proliferation of hepatocellular carcinoma cells [15]. Silencing of BCAT1 inhibits the tumorigenesis of ovarian cancer [32]. Notably, Xu et al. have demonstrated that overexpression of BCAT1 indicates a poor survival of GC and may serve as a diagnostic and therapeutic biomarker [19]. BCAT1 usually exerts its function in regulating the tumorigenesis as a downstream target of miRNAs [34, 35]. In this study, BCAT1 was determined to be a target of miR-3200-5p, which was negatively regulated by miR-3200-5p. Based on the above previous findings, we speculate that miR-3200-5p may inhibit the progression of GC by downregulating BCAT1. Additionally, we also found that the expression of BCAT1 was positively related to LINC00324 in GC tissues. Given that miR-3200-5p was a target of LINC00324, we assumed that LINC00324 may regulate BCAT1 through binding to miR-3200-5p. Encouragingly, the following rescue experiments displayed that si-LINC00324 reversed the promoting effects of BCAT1 on the proliferation, migration, and invasion of GC cells. To sum up, we speculate that silencing of LINC00324 attenuates the tumorigenesis of GC through regulating the miR-3200-5p/BCAT1 axis.

## 5. Conclusions

In conclusion, the expression of LINC00324 was upregulated in GC tissues and cells. Silencing of LINC00324 inhibited the proliferation, migration, and invasion of GC cells through regulating the miR-3200-5p/BCAT1 axis. Our research indicated that LINC00324 might act as a potential therapeutic target for GC. However, the detailed action mechanisms of LINC00324 on GC remain limited, and further researches are still needed.

## Figures and Tables

**Table 1:** Primer sequences used in quantitative real-time polymerase chain reaction (qRT-PCR).

Name of primer	Sequences (5′-3′)
LINC00324-F	CCCCCAGGAACTCCTTACTC
LINC00324-R	TGTGTCCTAGGGACGAAGGA
GAPDH-F	GTCGATGGCTAGTCGTAGCATCGAT
GAPDH-R	TGCTAGCTGGCATGCCCGATCGATC
miR-3200-5p-F	AAUCUGAGAAGGCGCACAAGGU
miR-3200-5p-R	TGGTGTCGTGGAGTCG
U6-F	CTCGCTTCGGCAGCACA
U6-R	AACGCTTCACGAATTTGCGT
BCAT1-F	CCAAAGCCCTGCTCTTTGTA
BCAT1-R	TGGAGGAGTTGCCAGTTCTT
*β*-Actin-F	ACACCTTCTACAATGAGCTG
*β*-Actin-R	CTGCTTGCTGATCCACATCT

**Table 2:** The relationship between the expression of LINC00324 and clinical characteristics in GC patients.

Variable	Total	LINC00324 expression		*P* value
		Low	High	
Age				0.605
<50	32	15	17	
≥50	28	15	13	
Gender				0.069
Male	33	20	13	
Female	27	10	17	
Tumor size				0.035^∗^
<5 cm	24	16	8	
≥5 cm	36	14	22	
Lymphatic metastasis				0.010^∗^
Yes	30	10	20	
No	30	20	10	
TNM stage				0.0014^∗∗^
I/II	32	22	10	
III/IV	28	8	20	
Histological grade				0.292
I/II	24	10	14	
III/IV	36	20	16	

## Data Availability

All data are available through the responsible corresponding author.
